# Psychological Impact and Quality of Life in Patients With Intestinal Stoma in Resource-Limited Settings: A Cross-Sectional Study From Pakistan

**DOI:** 10.7759/cureus.88292

**Published:** 2025-07-19

**Authors:** Rija Latifee, Faizan K Saleem, Tauqeer Ansari, Muhammad A Khan, Shah Muhammad

**Affiliations:** 1 General Surgery, Sindh Institute of Urology and Transplantation, Karachi, PAK; 2 Hepato-Pancreato-Biliary Surgery, Sindh Institute of Urology and Transplantation, Karachi, PAK; 3 General Surgery, Sindh Institute of Medical Sciences, Karachi, PAK; 4 Transplant Surgery, Sindh Institute of Urology and Transplantation, Karachi, PAK

**Keywords:** depression, intestinal stoma, pakistan, phq-9, psychosocial adaptation, quality of life

## Abstract

Background: Intestinal stoma formation alters patient lifestyle and psychological well-being, especially in resource-limited settings lacking perioperative support. This study assessed the quality of life (QoL) and prevalence of depression in Pakistani patients with stomas, identifying key demographic and clinical correlates.

Methods: A descriptive cross-sectional study was conducted among 136 patients with intestinal stomas at a tertiary care surgical clinic in Karachi. Structured interviews were conducted using a five-part questionnaire assessing demographics, stoma-related experiences, impact on daily life, QoL (7-45 scale), and depression (PHQ-9, Urdu-translated). Associations between variables were analyzed using chi-square tests.

Results: Most participants were male (73.5%) and married (80.9%). Temporary stomas were present in 66.9%, with 52.9% formed during emergency surgery. Only 25.7% received preoperative counseling. QoL scores were rated as "best" in 41.9% of participants, "good" in 55.9% of participants, and "poor" in 2.2% of participants. Marital status (p<0.001), employment (p=0.006), stoma permanence (p=0.036), and time since surgery (p=0.009) were significantly associated with QoL. Depression was absent in 41.2% of participants, mild in 39.7% of participants, and moderate to moderately severe in 19.1% of participants. Depression correlated with marital status, occupation, stoma type, and surgical urgency.

Conclusion: Most stoma patients adapt well over time, particularly with stable social and occupational conditions. However, gaps in preoperative counseling and somatic presentations of depression highlight the need for structured psychosocial support and culturally adapted tools.

## Introduction

The formation of a stoma, whether temporary or permanent, introduces profound changes to a patient’s daily life. While intended as a lifesaving or quality-preserving intervention for conditions such as colorectal malignancies, inflammatory bowel disease, or trauma, the postoperative experience is shaped not only by the underlying disease but also by the psychological and functional adjustments required to live with a stoma [1,2]. In low-resource settings, these challenges are amplified by limited access to specialized perioperative counseling, peer support, and stoma care infrastructure, potentially affecting adaptation and mental well-being.

Existing literature highlights that stoma patients face complex psychological and social challenges. Depression and anxiety are prevalent, with depression affecting up to 70% of patients in some studies [2,3]. In South Asian populations, poor access to counseling and the absence of stoma nurse interventions further exacerbate psychosocial distress and interfere with activities of daily living [4-6]. Several studies show that longer time since surgery leads to better adaptation, but the early postoperative period remains a window of vulnerability [7-10].

This study aimed to assess how Pakistani patients with intestinal stomas experience changes in daily function, emotional state, and social roles. Using structured interviews, we explored the relationship between stoma type, surgical context, and demographic factors on QoL and depressive symptoms. By focusing on lived experiences, we sought to identify patient subsets at higher risk for poor adaptation or psychological morbidity.

## Materials and methods

A descriptive cross-sectional study was conducted at the outpatient general and colorectal surgery clinics at Sindh Institute of Urology and Transplantation (SIUT), Karachi, from January to December 2024. Ethical approval was granted by the Institutional Review Board (Ref: SIUT/IRB/2023/078). A total of 136 consenting adult patients (≥18 years) with either temporary or permanent stomas (ileostomy or colostomy) were enrolled. Patients with cognitive impairment, psychiatric diagnoses, or communication disorders were excluded. The sample size was determined to detect a medium effect size (Cohen’s w = 0.3) with 80% power at a 5% significance level.

Data collection

Participants were interviewed by the primary investigator using a structured, pre-tested questionnaire developed for the study. The instrument comprised five sections: (i) Demographic and clinical characteristics: age, sex, education level, employment status, marital status, underlying disease, type and duration of stoma, and surgical context (elective/emergency); (ii) Stoma-related experience and adaptation: including exposure to counseling, prior knowledge of stoma, and subjective perceptions of adjustment; (iii) Impact on daily activities: assessed through patient-reported interference with mobility, social interactions, religious practices, and personal privacy (graded as “no effect,” “mild,” “moderate,” or “severe.” -please refer to Table 1); (iv) Quality of life (QoL): assessed using a validated QoL scale [11] with scores ranging from 7 to 45, (categorized as poor (7-26), good (27-45), and best (>45)); (v) Depression screening: using the Patient Health Questionnaire-9 (PHQ-9) [12], with severity interpreted per standard thresholds (0-4: no depression, 5-9: mild, 10-14: moderate, 15-19: moderately severe, 20-27: severe). To ensure data confidentiality, all responses were anonymized and coded. Data verification and integrity were maintained through double entry and secure digital storage.

**Table 1:** Grading Criteria for Impact on Daily Activities

Grade	Description						
No Effect	No noticeable impact on routine or psychological stress.						
Mild	Minor inconvenience or occasional emotional discomfort, with no change in routine.						
Moderate	Frequent disruption requiring adaptation or modification of specific activities.						
Severe	Major limitations necessitating withdrawal from usual roles or significant daily function.						

Statistical analysis

IBM SPSS Statistics for Windows, Version 27 (Released 2020; IBM Corp., Armonk, New York, United States) was used. Continuous variables were summarized as means±SD, categorical data as frequencies (%). Associations with QoL and depression were analyzed using chi-square tests. A p-value <0.05 was considered statistically significant.

## Results

Participant characteristics

A total of 136 patients who had undergone intestinal stoma formation were enrolled in the study. As outlined in Table 2, the cohort was predominantly male (73.5%) and married (80.9%). Most stomas were created for non-neoplastic conditions (77.2%) and were temporary in nature (66.9%), with colostomy being the predominant type (72.8%). Emergency surgeries constituted 52.9% of procedures, while the remaining were elective. One hundred thirty subjects had their stomas made one year ago, while four had them 6-9 months ago. Significance was noted between the educational level and the level of depression as out of 136 patients, the majority were of the illiterate variety (46%) who demonstrated mild or no level of depression, while the higher education ones (31%) had poor coping abilities, resulting in a higher level of depression (p-value less than 0.05).

**Table 2:** Demographic Characteristics of Respondents (N = 136)

Variable	Category	Frequency (n)	Percentage (%)
Sex	Male	100	73.5
	Female	36	26.5
Marital Status	Married	110	80.9
	Not Married	26	19.1
Underlying Disease	Malignant	105	77.2
	Benign	26	19.1
	Undefined	5	3.7
Nature of Stoma	Temporary	91	66.9
	Permanent	45	33.1

The subjects who followed up with us in our clinic mostly turned out to be male population which for our study is a limitation since the gender associations with QoL and the level of depression could not be compared due to unequal numbers. Despite the limited number of female patients included in our study (26.5%), out of 36 female patients, the majority (17) reported a mild level of depression. A similar case applies with the type of stomas that presented to us at the timeline of our data collection which mostly included temporary stomas and again the rightful significance and correlation couldn't be made between the two separate groups individually. However from 66.9% of temporary stomas, a significant correlation with the level of depression was noted as 42 individuals reported mild depression in association with temporary stoma.

Preoperative exposure and stoma adjustment

Patients demonstrated limited exposure to stoma-related information or peer support before surgery. Notably, 89% had not met another person with a stoma before their own operation, and the majority (58%) had not received satisfactory education about stoma management prior to surgery. A vast majority (88.2%) felt that meeting a stoma nurse prior to surgery would have improved their adjustment, underscoring a significant gap in preoperative counseling services.

Patient-reported impact on daily living

Analysis of lifestyle and psychosocial domains revealed a nuanced impact of stoma on daily life. Approximately half of the respondents reported no impact of their stoma on key domains such as anxiety, participation in social gatherings, privacy, or religious practices. However, a substantial proportion indicated intermittent or context-dependent distress. For instance, over one-third of participants expressed concern that their bag might open in public or emit odors, and around one-quarter frequently checked for accessible toilets due to fear of urgency or leakage. Sleep quality appeared to be a concern, with 69% reporting the need for daytime rest and 19.9% explicitly attributing this to nocturnal disturbances.

The effect on intimacy was heterogeneous; although 62% reported no change in spousal relationships, a meaningful subset experienced discomfort or strain. These findings suggest that while many individuals adapt effectively, others face persistent challenges that merit targeted psychosocial support.

QoL outcomes

QoL scores, as categorized into poor, good, and best functioning, indicated generally favorable adaptation among participants; 41.9% of individuals rated their QoL as "best," and 55.9% reported it as "good," leaving only 2.2% in the "poor" category.

When stratified by clinical and demographic variables, the chi-square test was performed, which showed significant differences in QoL. Specifically, marital status (Χ²=16.3, p < 0.001), employment status (Χ²=10.32, p = 0.006), the nature of the stoma (temporary vs. permanent; Χ²=6.52, p = 0.036), and the time elapsed since surgery (p = 0.009) were significantly associated with QoL scores, as detailed in Table 3. Our study included the time period since surgery starting from the first month and ending till the first year of surgery broken into four groups as follows: 1-3 months, 3-6 months, 6-9 months, and lastly greater than equal to one year past surgery; out of these, in the past one year 55 individuals reported no depression while only 10 subjects reported severe depression.

**Table 3:** Association Between Demographic and Clinical Variables and Quality of Life

Variable	Chi-square	df	p-value
Sex (Male vs Female)	3.12	2	0.211
Marital Status (Married vs Non-married)	16.3	2	0.001
Employment Status (Employed vs Unemployed)	10.32	2	0.006
Underlying Disease (Malignant vs Benign)	6.07	4	0.194
Nature of Stoma (Temporary vs Permanent)	6.52	2	0.036

These findings suggest that social support, financial independence, and time for adaptation may buffer against negative QoL impacts. Social support which included particularly the emotional and more specifically the financial support provided by the institute to the patients had a major role to play in their adaptation; patients had 24/7 access to get their problems regarding their stoma to be sorted whenever needed in addition to being provided with a spare stoma bag in case the present one got into some sort of non-functionality.

Depression scores and PHQ-9 trends

Depression severity, as assessed by the PHQ-9 scale, showed that 41.2% of participants had no depressive symptoms, while 39.7% experienced mild depression. A smaller proportion of patients were categorized as having moderate (11.0%) or moderately severe (8.1%) depression. These frequencies are presented in Table 4. Suicidal ideation was infrequently reported.

**Table 4:** Distribution of Depression Levels (PHQ-9) PHQ-9: Patient Health Questionnaire-9

Depression Level	Frequency	Percentage
No Depression	56	41.2%
Mild	54	39.7%
Moderate	15	11.0%
Moderately Severe	11	8.1%

**Figure 1:**
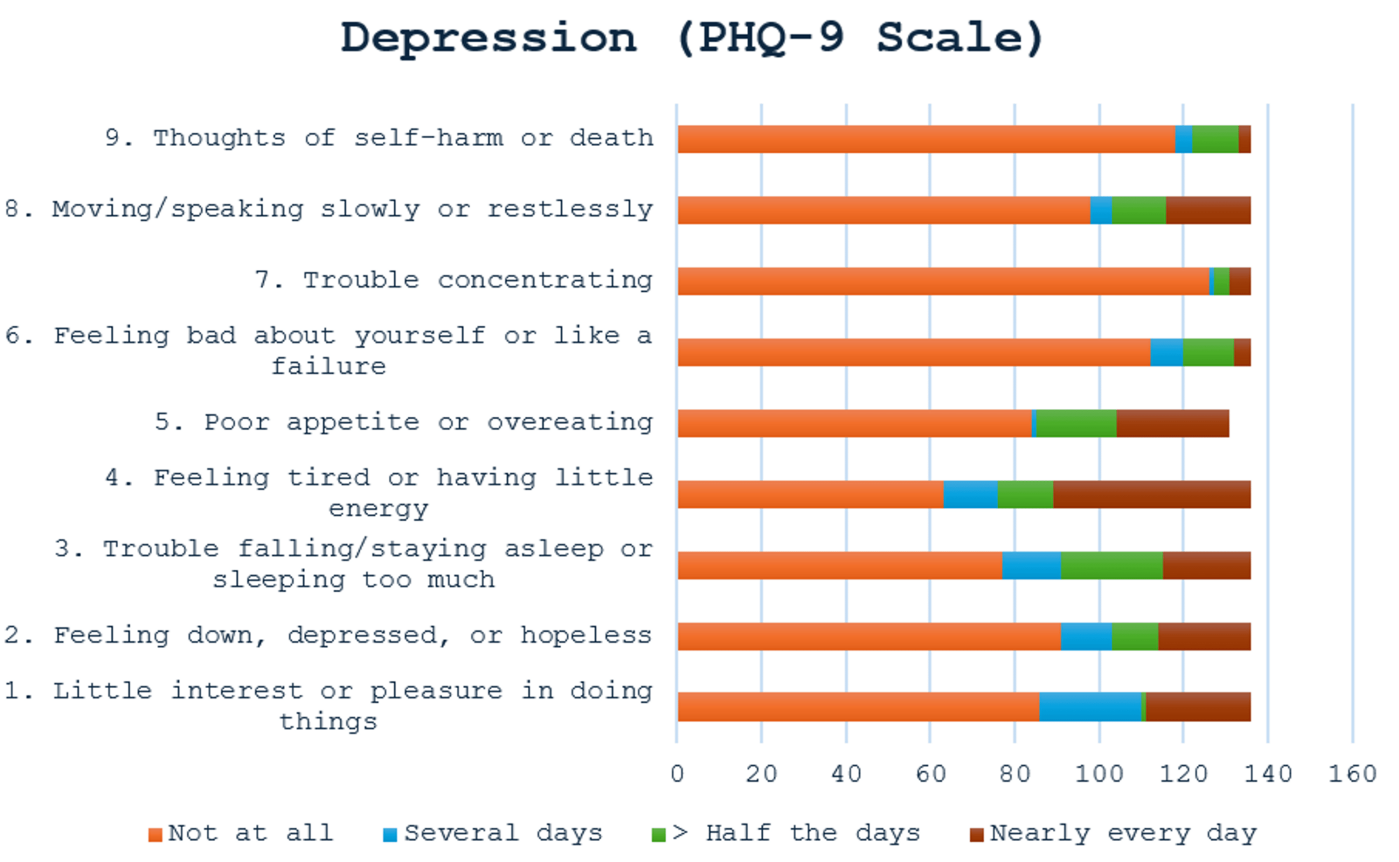
Likert-Scale Responses for Each Item on the PHQ-9 Depression Inventory Somatic symptoms such as fatigue, poor appetite, and sleep disturbances were more frequently reported, while cognitive and emotional symptoms such as hopelessness and suicidal ideation were less commonly endorsed. PHQ-9: Patient Health Questionnaire-9

Item-specific responses on the PHQ-9 revealed important patterns in depressive symptomatology. As shown in Figure 1, the most endorsed symptoms were fatigue, disturbed sleep, and poor appetite, each scoring higher on average than affective symptoms such as anhedonia or hopelessness. Meanwhile, cognitive-affective items, such as poor concentration and thoughts of self-harm, received the lowest average scores, suggesting that somatic complaints were more salient than mood-related symptoms in this population. This pattern reinforces the need to consider somatic presentations when screening for depression in stoma patients.

In analyzing determinants of depression severity, a chi-square test was performed, which showed several demographic and clinical characteristics to be statistically significant in associations with QoL and PHQ-9 category. As shown in Table 5, depression levels were significantly associated with marital status (Χ²= 9.42, p = 0.024), occupation (Χ²=7.92, p = 0.048), type of underlying disease (Χ²=23.76, p = 0.001), nature of the stoma (Χ²=15.67, p = 0.001), type of surgery (Χ²=33.61, p < 0.001), education (X²=56.91, p<0.001) and stoma type (Χ²=7.72, p = 0.052). Patients with temporary stomas, those undergoing emergency procedures, and those without structured preoperative counselling were more likely to report moderate to severe depressive symptoms. Conversely, patients with neoplastic disease appeared to report lower levels of depression, which may reflect either a more structured care environment or psychological relief following curative surgery.

**Table 5:** Association Between Demographic and Clinical Variables and Level of Depression

Variable	Chi-square	df	p-value
Marital status	9.42	3	0.024
Occupation	7.92	3	0.048
Underlying disease	23.76	6	0.001
Nature of stoma	15.67	3	0.001
Type of surgery	33.61	6	<0.001
Type of stoma	7.72	3	0.052
Level of education	56.91	12	<0.001

Summary of associations

The association analysis underscores the multidimensional factors influencing post-stoma adaptation. While gender couldn't be used to find significant association with either QoL or depression levels due to unequal distribution among the two genders which is a limitation of this study, social determinants such as marital and employment status played a clear role. Furthermore, longer time since stoma formation emerged as a protective factor for both outcomes, suggesting psychological adjustment over time.

## Discussion

This study reveals that Pakistani stoma patients, despite systemic limitations, generally adapt well, especially if married, employed, and post-op >1 year. Gender is a confounding factor in our study. However, many face early adaptation difficulties due to a lack of counselling, sleep disturbances, and somatic symptoms of depression.

Most participants had stomas for non-neoplastic conditions, consistent with low- and middle-income country (LMIC) literature where infectious or inflammatory diseases dominate surgical indications [4,5,13]. Patients with cancer-related stomas often viewed them as part of curative treatment, aiding psychological acceptance. According to a similar Pakistani study conducted by Irshad et al., most of their permanent stoma patients showed overall satisfaction with their life, except for half of the patients who needed financial, psychological, and relative support [4].

The absence of structured preoperative counselling and peer exposure aligns with prior studies [6,14,15]. All these studies highlight the importance of healthcare workers investing their time in researching new and more interventions to meet the psychological demands of the patients, which would ultimately help patients cope with the stresses of a stoma. Being able to relate to others with a stoma helps the participants to feel normal and ultimately accepted by others. It seems likely that being part of a community of people who have a similar experience contributes positively to stoma adjustment [14]. This point is important as our study revealed that out of 136 participants, the majority, that is 121 individuals, had never met anyone with a stoma before and hence couldn’t gather support and experience from another peer who had gone through all the adjustments and adaptations of that process.

Psychosocial interventions and pre-surgical stoma nurse visits significantly reduce stigma, anxiety, and social withdrawal. [1,12,16]. The presence of stoma complications has proven to have the most damaging effect on QoL, which agrees with research conducted worldwide. Patients report skin irritation in 50% of cases secondary to leakage that is due to in correct way of placing stoma and its associated appliances most of the time, it is sometimes secondary to incorrect position of the stoma that could have been correctly sited pre operatively if a stoma dedicated nurse was introduced to the patients, also the basic care needed to look after a stoma and its hygiene is where again a stoma dedicated nurse comes to play, our individuals pointed this out much clearly once asked, 120 (88.2%) subjects agreed that life would have been much easier if a nurse was provided to them in the initial time of the stoma process to cope through the whole procedure easily [5].

Before ostomy surgery, consideration of the stoma must be kept in mind. A customized plan of care must be created for the future ostomate to adapt so that it can be a coping mechanism for the individual and doesn’t come as a shock for the patient who is to undergo major changes in the life ahead [13], our study again highlighted the fact only 35 individuals were the fortunate ones who were given prior information and counselling regarding the process and possibility of stoma for which once they were operated were prepared for the challenges they were to face with the life bearing a stoma. They fell into the category of no or little depression.

Stoma produces social, domestic, and psychological upsets. Anxiety and embarrassment over a stoma may alter lifestyle, activities of daily living, including the ability to work, desire to travel, and overall self-image. The way patients feel about the changes in their bodies can affect their behavior toward family and friends. Some patients have initial problems with diet and clothing as well [3], similar traits were noted in our subjects to variable extent, though major pointed to the fact that stoma had ‘no effect’ on their level of anxiety, activities of daily living, their freedom of going out in public and attending different gatherings and clothing options.

Several areas can influence QoL for patients with an ostomy, such as ostomy management difficulties, access to medical coverage, and adequate reimbursement for services and supplies. An important consideration is related to the financial burden of paying for ostomy supplies that can influence the QoL. Our patients after stoma formation are given 24/7 access to get their troubles regarding stoma to be sorted, which includes simply reapplying the stoma bag, dealing with stoma-related skin irritations and complications such as prolapsed or retracted or swollen/edematous stomas and also providing our patients with one spare stoma bag and its appliances if in case the other gets into some technical difficulties. Negative effects are frequently associated with ostomy complications and often lead to life limitations in either physical or psychosocial areas for individuals and their families [1], these points were highlighted in our study by several questions concerning the bag, its filling up, its emptying for which individual required a nearby toilet, the noises coming out of their stoma bag and its associated odor that contributed to individual's social withdrawal and additional anxiety, out of our subjects, 25% reported getting worried when the bag was full, 35% were concerned about the bag opening up in public and resulting in embarrassment, 9.6% were always at a search to find a nearby toilet, 8% were anxious regarding bag smelling and 15% were disturbed by the sounds coming out of stoma.

Somatic symptom predominance in depression (sleep, appetite, fatigue) aligns with South Asian psychocultural norms [17,18]. Mild depressive symptoms were common, but cognitive-affective features were less endorsed. This highlights the need for culturally sensitive screening and interpretation.

Our study has various limitations, firstly as mentioned previously the unequal distribution between both genders, which has a major role since a lot of studies conducted on similar subjects pointed out the female gender as the one showing major change in their QoL and level of depression as a result of a stoma [19]. This study has a limitation in being a cross-sectional study as the fact that these behavioral changes are all adaptive and resolves with time couldn't be executed, also our study had major subjects who had temporary stomas so the true adaptation as noted for those patients who have permanent stoma couldn't be explored which we believe is the actual variation that needs life-long adaptations and acceptance; nevertheless, it has also certain strengths like the valid tool with which the whole questionnaire was designed and translated in the native language and the structured way the study was conducted.

## Conclusions

This study highlights that psychosocial adaptation to stomas in Pakistan is heterogeneous but generally positive, particularly over time. Structured counseling, peer exposure, and early identification of depression are essential to improve outcomes. Integrating psychosocial support and stoma nurse education into surgical care pathways in LMICs is strongly recommended.

## Appendices

Questionnaire

https://docs.google.com/document/d/1S63kqftHybkUmqIVIBqoHHxqO2lc3HRm/edit?usp=drivesdk&ouid=114846327095202134869&rtpof=true&sd=true

## References

[1] Alenezi A, McGrath I, Kimpton A, Livesay K (2021). Quality of life among ostomy patients: a narrative literature review. J Clin Nurs.

[2] Farahani MA, Sargolzaei MS, Shariatpanahi S, Dehkordi AH, Dalvand P, Heidari-Beni F (2022). The prevalence of anxiety and depression in patients with ostomy: a systematic review and meta-analysis. Psychooncology.

[3] Musa RM, Salih MA, Shaaban KM, Abdelbagi AY (2021). Quality of life, levels of anxiety and depression among Sudanese patients with surgical stoma in Khartoum State, Sudan. Ann Afr Med.

[4] Irshad A, Ahsan MF, Khan MA, Rashid I, Aseef M (2021). Quality of life in patients with stoma. Ann PIMS.

[5] Hussain S, Dioso RI (2025). Quality of life in patients with permanent colostomy. Medtigo J Med.

[6] Silva NM, Santos MA, Rosado SR, Galvão CM, Sonobe HM (2017). Psychological aspects of patients with intestinal stoma: integrative review. Rev Lat Am Enfermagem.

[7] Zewude WC, Derese T, Suga Y, Teklewold B (2021). Quality of life in patients living with stoma. Ethiop J Health Sci.

[8] Temiz Z, Cavdar I, Ozbas A, Altunsoy M, Akyuz N, Kutlu FY (2022). Sleep quality and factors affecting sleep in individuals with an intestinal ostomy: a descriptive cross-sectional study. Wound Manag Prev.

[9] Vonk-Klaassen SM, de Vocht HM, den Ouden ME, Eddes EH, Schuurmans MJ (2016). Ostomy-related problems and their impact on quality of life of colorectal cancer ostomates: a systematic review. Qual Life Res.

[10] Petersén C, Carlsson E (2021). Life with a stoma-coping with daily life: experiences from focus group interviews. J Clin Nurs.

[11] Prieto L, Thorsen H, Juul K (2005). Development and validation of a quality of life questionnaire for patients with colostomy or ileostomy. Health Qual Life Outcomes.

[12] (2024). Patient Health Questionnaire-9 (PHQ-9). https://www.apa.org/depression-guideline/patient-health-questionnaire.pdf.

[13] Ullah N, Muhammad D, Shah M (2020). Experience of individual living with prolong ostomy: a qualitative study. J Pak Med Assoc.

[14] Kittscha J, Wilson V, Fairbrother G, Bliokas V (2024). The role of peer support groups in adjustment to stoma. Collegian.

[15] Avci Işik S, Balanuye B, Budak Ertürk E (2023). Sleep problems in individuals with intestinal stomas and determining the quality of sleep: a multicenter study. J Wound Ostomy Continence Nurs.

[16] Black P, Notter J (2021). Psychological issues affecting patients living with a stoma. Br J Nurs.

[17] Kirmayer LJ (2001). Cultural variations in the clinical presentation of depression and anxiety: implications for diagnosis and treatment. J Clin Psychiatry.

[18] Wang SM, Jiang JL, Li R (2024). Qualitative exploration of home life experiences and care needs among elderly patients with temporary intestinal stomas. World J Gastroenterol.

[19] Brady RR, Sheard D, Howard K (2025). The prevalence of leakage, peristomal skin complications and impact on quality of life in the first year following stoma surgery. Nurs Rep.

